# Evaluation of the Cleaning Procedure Efficacy in Prevention of Nosocomial Infections in Healthcare Facilities Using Cultural Method Associated with High Sensitivity Luminometer for ATP Detection

**DOI:** 10.3390/pathogens7030071

**Published:** 2018-08-31

**Authors:** Beatrice Casini, Benedetta Tuvo, Michele Totaro, Francesco Aquino, Angelo Baggiani, Gaetano Privitera

**Affiliations:** 1 Department of Translational Research, N.T.M.S., University of Pisa, 56123 Pisa, Italy; tuvobenedetta@hotmail.it (B.T.); micheleto@hotmail.it (M.T.); Angelo.baggiani@med.unipi.it (A.B.); gaetano.privitera@med.unipi.it (G.P.); 2 Department of Pubblic Health and Hygiene, Azienda USL Toscana Nord Ovest, 56124 Pontedera, Italy; cheps86@hotmail.it

**Keywords:** environmental surfaces, air quality, ATP bioluminometry

## Abstract

In healthcare facilities, environmental surfaces may be a reservoir of infectious agents even though cleaning and disinfection practices play a role in the control of healthcare-associated infections. In this study, the effectiveness of cleaning/disinfection procedures has been evaluated in two hospital areas, which have different risk category classifications. According to the contract with the cleaning service, after the daily ambulatory activities, the housekeeping staff apply an alcohol-based detergent followed by a chlorine-based disinfectant (2% Antisapril, Angelini; 540 mg/L active chlorine), properly diluted and sprayed. The contract provides for the use of disposable microfiber wipes which must be replaced with new ones in each health out-patient department. Surface contamination was analyzed using cultural methods and ATP detection, performed with a high-sensitivity luminometer. The values 100 CFU/cm^2^ and 40 RLU/cm^2^ were considered as the threshold values for medium-risk category areas, while 250 CFU/cm^2^ and 50 RLU/cm^2^ were defined for the low-risk category ones. Air quality was evaluated using active and passive sampling microbiological methods and particle count (0.3 μm–10 μm) detection. The cleaning/disinfection procedure reduced the medium bacterial counts from 32 ± 56 CFU/cm^2^ to 2 ± 3 CFU/cm^2^ in the low-risk area and from 25 ± 40 CFU/cm^2^ to 7 ± 11 CFU/cm^2^ in the medium-risk one. Sample numbers exceeding the threshold values decreased from 3% and 13% to 1% and 5%, respectively. RLU values also showed a reduction in the samples above the thresholds from 76% to 13% in the low-risk area. From the air samples collected using the active method, we observed a reduction of 60% in wound care and 53% in an ambulatory care visit. From the air samples collected using the passive method, we highlighted a 71.4% and 50% reduction in microbial contamination in the medium-risk area and in the low-risk one, respectively. The 10 μm size particle counts decreased by 52.7% in wound care and by 63% in the ambulatory care visit. Correct surface sanitation proved crucial for the reduction of microbial contamination in healthcare settings, and plays an important role in ensuring air quality in hospital settings.

## 1. Introduction

Routine cleaning practices are often suboptimal, with an increased likelihood of the presence of pathogens. The role the physical environment plays in the acquisition of healthcare-associated infections (HCAIs) is increasingly recognized. Many microbial pathogens can survive for weeks in the absence of decontamination [1,2].

The issue of patient infection risk through contaminated hard surfaces in hospital rooms has been widely discussed [3,4]. Patients are frequently subject to environmental nosocomial pathogens. Environments are frequently contaminated and may be a reservoir for the transmission of pathogens either directly through patient contact with the environment or indirectly through the contamination of healthcare workers’ hands and gloves [5].

Recent studies have investigated the benefits of better cleaning practices, including different methods, evaluating cleaning efficacies [6], the microbial burden on commonly touched surfaces [7], and the relationship with cleaning standards [8,9,10]. Although not validated, microbiologic standards for a safer hospital environment have been proposed as three colony-forming units (CFU)/cm^2^ on surfaces [6,11,12]; this value is related to an ATP value of 100 RLU/100cm^2^ [8,10,13]. Maintaining counts below these thresholds may assist in reducing HCAIs.

A recent review of intervention studies suggests that improvements in environmental disinfection may prevent the transmission of infectious agents and reduce HCAIs. Improved environmental cleaning resulted in decreased contamination and infection, as shown in a study of the spread of VRE in a 21-bed medical intensive care unit with high-level endemicity [14]. Aimed at evaluating sequential interventions of *C. difficile* isolation room disinfection in a Veterans Administration Hospital, increased cleaning and disinfection of high-touch surfaces led to decreased surface contamination [15].

In recent years, there has been a great deal of research into the mechanical removal capacity of dirt and microorganisms. Microfiber responds to the cleaning needs not only of high-touch surfaces but also of the environment, even reducing the chemical risk for workers, since its use reduces the consumption of chemical products.

Unlike traditional cloths, which act as a carrier for chemical detergents, microfiber has a mechanical action on dirt molecules, imprisoning them between the fibers associated with water use. The main features are capillarity, electrostaticity, and the failure to release particles.

One particularity of microfiber is that it does not release particles during use. For this reason, it is very effective even in the disinfection of the surfaces of premises, such as hospitals, where the issue of air contamination must be tackled. It also appears to be effective even after several cycles of reconditioning [16,17].

Several methods have been used to assess environmental cleanliness; one such method is the aerobic colony count (ACC) assay, which reveals the amounts of cultivable bacteria present on surfaces. The original quantitative ACC-based standard for defining the surfaces in a ward environment as clean was less than 5 CFU/cm^2^, but this value has been reduced to 3 CFU/cm^2^ [7,10,18].

Currently, the non-culture adenosine triphosphate (ATP) bioluminescence assay is extensively used to evaluate cleanliness because readings can be obtained on site. Because of its presence in living organisms, ATP was first used as an indicator of cleanliness in the food industry [19]. Subsequently, ATP measurements have been employed to assess hospital cleanliness using different benchmark values expressed in relative light units (RLUs). Quantitative results are available in less than 5 min with these assays. This makes it possible for infection prevention or housekeeping staff to monitor the adequacy of cleaned surfaces [6].

The microbial evaluation of surfaces is useful for monitoring the effectiveness of cleaning and disinfection practices. The aim of the study was to evaluate cleaning procedure efficacy in reducing bacterial contamination and the specific nosocomial multi-resistant pathogens, using cultural methods, associated with a high-sensitive luminometer for ATP detection.

## 2. Results

To estimate the value where the best performance of the luminometer test is obtained, the ROC (Receiver Operating Characteristic) curve has been constructed, choosing different threshold values. Therefore, the sensitivity and specificity of the test has been calculated, after setting a threshold that varies according to the type of clinic.

The values of 40 RLU/100 cm^2^ and 100 CFU/100 cm^2^ were chosen for the wound surgery and 50 RLU/100 cm^2^ and 250 CFU/100 cm^2^ for the ambulatory care visit. In the former case, specificity and sensitivity values were obtained with these limits (as reported by the literature by Mulvey et al., 2011 [10]) equal to 20% and 100%, respectively, while in the ambulatory care visit they amounted to 23% and 50%.

The mean RLU values recorded in the low-risk and medium-risk outpatient clinic, before cleaning, were 995.79 ± 1494 and 404.53 ± 474, respectively; after cleaning they were 462.09 ± 486 and 284.21 ± 336, respectively. In the first case, there is a percentage decrease equal to 59.4%, while in the second case this amounts to 38.5%. A high correlation was not underlined between the RLU and CFU values (r^2^ = 0.0018).

**Figure 1:**
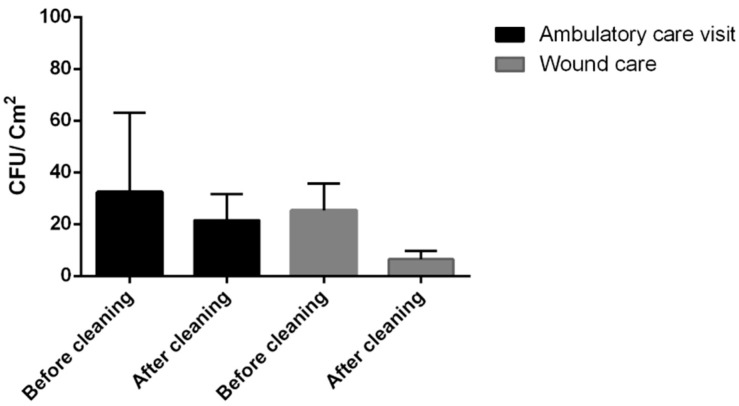
Mean and standard deviation of CFU values found before and after cleaning, on surfaces, in each ambulatory setting.

From a total of 560 samples analyzed, all the samples were negative for the research into *S. aureus*, *P. aeruginosa*, *Aspergillus* spp. and enterobacteria. We investigated these parameters according to the hygienic standards proposed by ISPESL 2009 guidelines. Before cleaning, the mean microbial values were 32 ± 56 CFU/cm^2^ in the low-risk area and 25 ± 40 CFU/cm^2^ for the low- and medium-risk environments. The percentages of samples that resulted over the threshold were 3% and 13%, respectively. After cleaning, the average values were: 2 ± 3 CFU/cm^2^ and 7 ± 11 CFU/cm^2^, with a reduction of the samples above the threshold at 1% and 5%, respectively. The reduction rates recorded were 92% for the ambulatory care unit and 98.3% for the wound care (Figure 1).

The analysis of the data recorded for the particulates demonstrated a reduction in all the classes of particles (Table 1), showing an average percentage decrease of 28.38%.

From the particle count detection, we observed a decrease in all the particle sizes, mostly for those with a size of 10 μm (Table 1 and Table 2).

The active air samples showed a 60% reduction in the total microbial counts at 37 °C, in wound care (from 45 ± 5 CFU/cm^2^ to 18 ± 12 CFU/cm^2^), and a 53% reduction in ambulatory care visit (from the mean values 34 ± 10 CFU/cm^2^ to 10 ± 8 CFU/cm^2^). The passive air samples showed a 71.4% reduction in the total microbial counts at 37 °C (from 7 ± 5 CFU/cm^3^ to 2 ± 1 CFU/cm^3^) in the low-risk area, and a 50% reduction (from the mean values 6 ± 1 CFU/cm^3^ to 3 ± 1 CFU/cm^3^) in the medium-risk one. Moreover, we detected a correlation between the reduction in the surface microbiological results and the particle counts (r^2^ = 1).

## 3. Discussion

The assessment of and feedback on cleaning performance is a critical part of environmental infection prevention. Traditionally, the monitoring of cleaning quality is performed through a visual inspection of the area [20]. Several studies have questioned the accuracy of visual inspections compared to both microbiological sampling methods and non-microbiological sampling methods [10,21,22]. The latter include fluorescent markers as surrogates of residual contamination, or the quantification of adenosine triphosphate (ATP) levels representing the persistence of organic material [23].

The luminometric method is simple to perform, can be done in the field, and can provide a quick indication of the degree of surface sanitization [24,25]. Our results cannot be considered as indicators of microbial contamination, considering also that ATP molecules present on surfaces may not have a microbial origin. Currently, ACCs of < 2.5–5 CFU per cm^2^ on hand-touch sites have been assigned as a microbiological limit [6,21]. An additional international institution also uses microbiological standards incorporating the presence of indicator organisms. Their identification depends on the risk to human health and on the matrix inspected [26,27].

ATP systems have varying benchmarks, depending on the type of luminometer used. These range from 25–500 RLUs for 10–100 cm^2^ on hospital surfaces [8,13]. A poor correlation between microbial contamination and RLU values has been demonstrated, where the values of the former are low, as in the case of the ambulatory care visit with a very low level of microbial contamination [28]. For this reason, the relationship between the two indices considered is not evident [29].

Different values should be chosen, depending on patient risk; surfaces in outpatient clinics are not necessarily as critical for infection risk as sites beside a ventilated patient receiving intensive care.

ATP can also be confounded by disinfectants (bleach), microfiber products, and manufactured plastics used in cleaning and laundering industries [8,13,30]. If an ATP assessment was introduced into hospitals, it should help to monitor cleaning quality and its failures, even when there is not a serious risk for patients.

In conclusion, since a visual assessment does not offer reliable information on infection risk to patients, high-risk (hand-touch) surfaces in hospitals should be subjected to a scientific screening method to monitor the overall levels of microbial dirt. If they were integrated into a formal monitoring regimen, ATP and/or microbiological benchmarks would help to identify unacceptable soil levels and associated patient risk provided they were systematically collected over time and interpreted accurately [7,13,31].

We evaluated indoor air quality in the ambulatory care visit and its correlation with the presence of cultivable bacteria.

Airborne particles come from multiple sources, of which the most relevant is the shedding of squames or skin scales. On average, an individual with a moderate level of physical activity sheds about 10 min^−1^ particles measuring at least 0.5 mm in diameter [32]. Despite their large size, squames circulate via the convection currents created by the temperature gradient between the body and the environment [33]. Other sources of airborne particles include dust and condensation droplets measuring less than 5 μm in diameter and representing the remnants of larger droplets produced during coughing, talking, and suction systems.

Particle size influences the tendency to settle on surfaces. Particles smaller than 5 μm remain suspended in the air, while those larger than 100 μm settle rapidly, and those of an intermediate size (5–100 μm) may settle on potentially contaminated surfaces and then migrate to other sites [34]. Particles may carry variable bacterial loads, depending on their source [35].

Both active and passive air sampling techniques can be used as a general monitor for air pollution, and for the purposes of routine surveillance programs. An air sampler measures the microbial burden more accurately. A settle plate is a direct indicator of SSI risk.

The selection of the method depended on the specific type of information we needed. If the sampling is performed to obtain information on the concentration of all inhalable viable particles, the active method should be preferred. On the contrary, if the air sampling performed during ambulatory activity is carried out to monitor the risk of microbial wound contamination, a passive measurement is better than volumetric sampling for predicting the likely contamination rate, as it allows for a direct measurement of the number of microorganisms settling on surfaces [36]. Hence, this economic and simple settle plate method has a more practical application in reflecting the risk of infection. The index of microbial air contamination IMA has proved to be a reliable and useful tool for monitoring the microbial surface contamination settling from the air in any environment. 

The data obtained in this study suggest that the improved cleaning program contributed to indoor air quality through the reduction of airborne dust mass and cultivable bacteria.

## 4. Materials and Methods

### 4.1. Healthcare Setting

In the period between January and July 2017, samplings were performed every three days. In the investigated hospital, in North-West Tuscany, Italy, only care activities do not provide the standard patient admission and urgent access. The various hospital activities, such as endocrinology, diabetology, oncology and ophthalmology, are applied over five floors. We chose two types of ambulatory activity with different risk classifications: A wound care ambulatory clinic and a diabetology ambulatory care visit. We chose these two types of ambulatory setting because they had a high patient turnover.

### 4.2. Cleaning Procedure

In this hospital, cleaning services are outsourced to an external company. According to the contract with the cleaning service, after the daily ambulatory activities, the housekeeping staff apply an alcohol-based detergent followed by a chlorine-based disinfectant (2% Antisapril, Angelini; 540 mg/L active chlorine), properly diluted and sprayed. The contract provides for the use of disposable microfiber wipes which must be replaced with new ones in each ambulatory setting.

### 4.3. Sample Collection

During the study period, 560 surface samples were collected, evaluating microbial contamination using a cultural method and through the application of a high-sensitivity bioluminometer. Moreover, a further 112 and 336 air samples, gathered using the active and passive method, were collected, respectively. At the same time, a particle count (0.3–10 µm) detection were performed. All the samples were collected one hour before and one hour after the cleaning/disinfection procedure. The number of samples collected over a 6-month monitoring period during the study was selected to be representative of the average microbial contamination on surfaces, considering the potential variability of healthcare activity during a year.

### 4.4. Microbiological and Particle Count Analysis

To evaluate surface cleanliness, both the standard stamp agar method (stamp method) and the adenosine triphosphate (ATP) bioluminescence for ATP detection were applied in this study, as described by Shimoda et al., 2015 [37].

To detect the microbial contamination from cultivable bacteria, 3 swabs (APTACA, Cannelli, Italia) were collected, in accordance with ISO 14698-1; one for monitoring viable bacteria growth (PCA, Merck Millipore), one for the isolation of *S. aureus* (Mannitol Salt Agar, Merck Millipore), and one for enterobacter detection (MacConkey agar, Merck Millipore). The samplings were performed on each 10 × 10 cm high touch surface (on a desk, briefcases, a bathroom handle and door, and a bookcase), using a sterile swab moistened in Ringer Solution (Oxoid, Hampshire, UK). Afterwards, swabs were placed in well-labelled swab caps and transported back to the laboratory immediately, as mentioned above.

At the same time, before and after the daily cleaning procedure, the same surface samples were analyzed for ATP detection using an ATP bioluminescence kit purchased from 3M (3M Clean-Trace ATP System; 3M Co., St. Paul, MN, USA). ATP swabs were taken from fully dried surfaces of areas immediately adjacent to the areas sampled for the culture assays. After sampling, the ATP swabs were placed in ATP bioluminescence reaction tubes and agitated to allow the reaction to occur. After this, the reaction tubes were inserted into a luminometer, and ATP readings were obtained and expressed in RLU.

Passive air samplings were performed using Plate Count Agar (PCA) plates (Oxoid, Basingstoke, UK), placed in three different room sites, (on a desk, a meeting table, and a bed) and exposed for 1 h, as described by Montagna et al. 2017 [38].

Active air samplings were carried out according to UNI EN ISO 14689, using the Surface Air System (SAS) (PBI International, Rockville, MD, USA). The device has been approved by the US Food and Drug Administration, the American Conference of Governmental Industrial Hygienists, and the American Society for Testing and Materials Committee—USP 23-NF 18–8th Supplement (May 1998)—Microbiological Evaluation of Clean Rooms and Other Controlled Environments—European Union Guide for GMP—Manufacture of Sterile Medicinal Products Control of Medicines and Inspection and CEN/TC 243 Norms for Clean Room Technology.

Five hundred liters of air was aspirated at a fixed speed (120 L/min) for a variable time through a cover which had been machined with a series of small holes with a special design.

The resulting laminar air flow was directed onto the agar surface. When the pre-set sampling cycle was complete, the PCA was removed and incubated at 37 °C for 24 h, to detect the total microbial count at 37 °C. For each plate, the calculation of the CFU/m^3^ was obtained as follows: CFU/m^3^ = (MPN/plate × 1000)/air volume (L); following the manufacturing instructions. 

Particle counts (0.3–10 μm) were monitored in both ambulatory environments. In each setting, we sampled two points: the center, on a meeting table, and the one corners of the room, on a patient’s bed. Particle counts samplings were performed by the Hach Met One 3313 Particle Counter (Ashtead Technology, Aberdeen, UK).

## Figures and Tables

**Table 1:** Mean and standard deviations for each class of particles, before and after cleaning, for wound care.

	0.3 µm	0.5 µm	1 µm	3 µm	5 µm	10 µm
	Mean ± SD	Mean ± SD	Mean ± SD	Mean ± SD	Mean ± SD	Mean ± SD
**BEFORE CLEANING**	14 × 10^6^ ± 12 × 10^6^	94 × 10^4^ ± 12 × 10^4^	34 × 10^4^ ± 52 × 10^3^	10 × 10^4^ ± 17 × 10^4^	28 × 10^3^ ± 13 × 10^3^	54 × 10^2^ ± 19 × 10^2^
**AFTER CLEANING**	13 × 10^6^ ± 10 × 10^6^	87 × 10^4^ ± 25 × 10^3^	29 × 10^4^ ± 99 × 10^2^	49 × 10^3^ ± 11 × 10^3^	17 × 10^3^ ± 67 × 10^3^	20 × 10^2^ ± 38 × 10
**% VARIATION**	Decrease 8.3%	Decrease 7.2%	Decrease 14.5%	Decrease 46.5%	Decrease 41.1%	Decrease 63%

**Table 2:** Mean and standard deviations for each class of particles, before and after cleaning, for ambulatory care visit.

	0.3 µm	0.5 µm	1 µm	3 µm	5 µm	10 µm
	Mean ± SD	Mean ± SD	Mean ± SD	Mean ± SD	Mean ± SD	Mean ± SD
**BEFORE CLEANING**	16 × 10^6^ ± 14 × 10^6^	88 × 10^4^ ± 6 × 10^4^	30 × 10^4^ ± 48 × 10^3^	6 × 10^4^ ± 10 × 10^4^	18 × 10^3^ ± 10 × 10^3^	63 × 10^2^ ± 29 × 10^2^
**AFTER CLEANING**	15 × 10^6^ ± 11 × 10^6^	77 × 10^4^ ± 25 × 10^3^	27 × 10^4^ ± 96 × 10^2^	40 × 10^3^ ± 9 × 10^3^	13 × 10^3^ ± 55 × 10^3^	29 × 10^2^ ± 37 × 10
**% VARIATION**	Decrease 6.2%	Decrease 12.5%	Decrease 10%	Decrease 3.3%	Decrease 27.8%	Decrease 52.7%
